# Conversion therapy for advanced intrahepatic cholangiocarcinoma with lenvatinib and pembrolizumab combined with gemcitabine plus cisplatin: A case report and literature review

**DOI:** 10.3389/fimmu.2022.1079342

**Published:** 2023-01-09

**Authors:** Wei Zhang, Chu Luo, Zun-Yi Zhang, Bi-Xiang Zhang, Xiao-Ping Chen

**Affiliations:** Hepatic Surgery Center, Tongji Hospital, Tongji Medical College, Huazhong University of Science and Technology, Wuhan, China

**Keywords:** intrahepatic cholangiocarcinoma, immunotherapy, antiangiogenic therapy, chemotherapy, conversion therapy

## Abstract

**Background:**

Intrahepatic cholangiocarcinoma (ICC) is a highly malignant biliary tumor. Patients with unresectable and advanced ICC have a poor prognosis with current gemcitabine-based chemotherapy. Combination therapy strategies based on immunotherapy have achieved promising results in various tumor types.

**Case presentation:**

We reported a patient with unresectable ICC who received lenvatinib and pembrolizumab in combination with gemcitabine plus cisplatin (GP) chemotherapy and subsequently underwent radical liver resection. A 46-year-old male with a history of chronic hepatitis B and hypertension was diagnosed with ICC. Multiple liver tumors with ring-like enhancement were detected on abdominal contrast-enhanced CT and MRI. Enlarged lymph nodes were found in the hilar and retroperitoneal areas. The tumor was clinically staged as T2N1M0 (stage IIIB). Lenvatinib and pembrolizumab in combination with GP chemotherapy were adopted as first-line treatments for the patient. After six cycles of scheduled treatment, the diameter of the largest liver lesion and the number of liver lesions were markedly reduced. The level of the tumor marker CA19-9 decreased to a normal range. A partial response according to the mRECIST criteria was achieved without severe toxicities. Non-anatomical liver resection (segment 4b, 5,6 + segment 7 + segment 8), cholecystectomy and hilar lymph node dissection were performed one month after stopping combination therapy. Pathological examination confirmed a diagnosis of moderate-to-poorly differentiated ICC with lymph node metastasis. The patient has survived 15 months following resection of the tumors, with no evidence of local recurrence or distant metastasis.

**Conclusion:**

Lenvatinib and anti-PD1 antibody pembrolizumab in combination with GP chemotherapy provided promising antitumor efficacy with reasonable tolerability, which may be a potentially feasible and safe conversion therapy strategy for patients with initially unresectable and advanced ICC.

## Introduction

Cholangiocarcinoma is a heterogeneous group of malignant epithelial tumors arising from the biliary epithelium (1). Cholangiocarcinoma can be divided into intrahepatic cholangiocarcinoma (ICC) and extrahepatic cholangiocarcinoma (including hilar and distal bile duct) (2). The incidence and mortality of ICC have increased during the last three decades (3). To date, the most effective method of treating ICC is early radical surgical removal. However, most patients are in the advanced stage when diagnosed with ICC and lose the opportunity for surgical treatment (4). The median survival time of patients with advanced cholangiocarcinoma is less than two years, and the five-year survival rate is only 10% (5). Systemic conventional chemotherapy with gemcitabine and cisplatin (GP) is the standard first-line treatment, although the prognosis remains poor with a median survival of less than one year (6). Existing treatment options have limited and variable efficacy. Thus, it is urgent to develop novel effective therapies for ICC.

Recently, due to the development of precision medicine, immunotherapy has played an important role in treating tumors by preventing escape from immune surveillance. Immune checkpoint inhibitors (ICIs) have been successfully used to treat malignancies such as hepatocellular carcinoma (HCC), leading to an increasing number of studies evaluating their use in biliary tract cancer (BTC) (7, 8). Although these new inhibitors have improved patient survival, the effectiveness of ICIs monotherapy remains relatively limited, achieving an objective response rate (ORR) of up to approximately 20% (9). Numerous trials are being conducted to investigate therapy strategies using combinations of ICIs with immunotherapy, chemotherapy, anti-angiogenics or local therapy, with preliminary results showing promising antitumor efficacy and tolerable safety (10–12). Lenvatinib, a novel oral multi-kinase inhibitor, exerts both antiangiogenic and direct antitumor effects by targeting multiple kinase receptors, including vascular endothelial growth factor (VEGF), fibroblast growth factor (FGF), and platelet-derived growth factor (PDGF) receptors (13). Recently, the combination of anti-PD1 antibody with lenvatinib has shown remarkable results in several retrospective studies included patients with advanced BTC (14, 15).

Here, we report a patient with unresectable and advanced ICC who received successful radical surgical resection after combination therapy with lenvatinib and the anti-PD1 antibody pembrolizumab combined with gemcitabine plus cisplatin chemotherapy as first-line treatment.

## Case presentation

**Figure 1:**
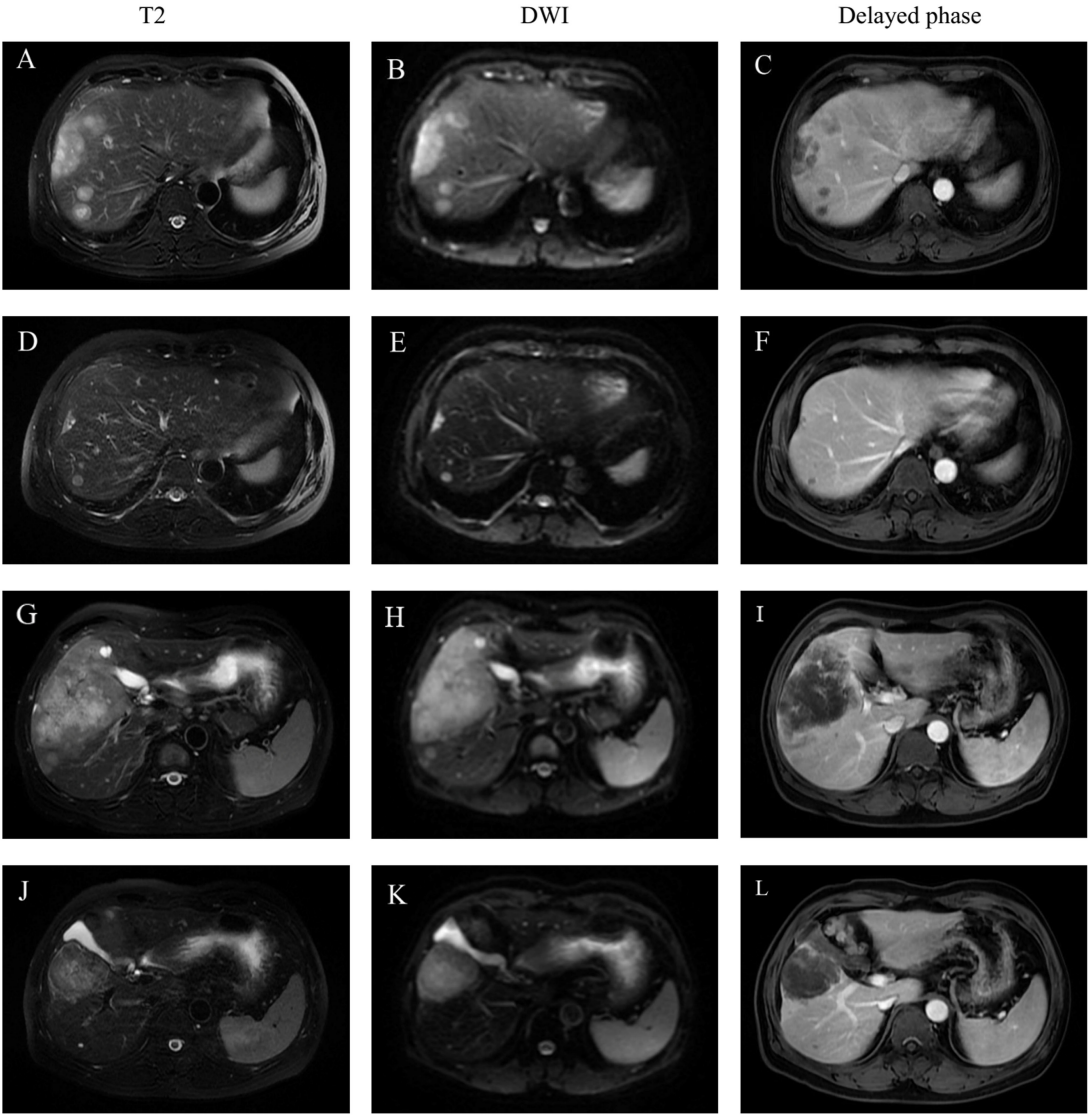
Representative MR imaging revealed the changes of multiple lesions before and after combination treatment. **(A, D, G, J)**: An axial T2-weighted MR image. **(B, E, H, K)**: An axial diffusion weighted image (DWI). **(C, F, I, L)**: An axial delayed phase images. **(A, B, C**: A dynamic contrast-enhanced MRI showed that multiple liver tumor located in the segment 7 and 8 before treatment. **(D, E, F**: MRI results after six cycles of treatment revealed that the tumor located in the segment 7 and 8 has shrunk in diameter and the number of tumors decreased. **(G, H, I)**: MRI showed the largest tumor (measuring 11.5 cm in diameter) located in the right anterior lobe and segment 6 before treatment. **(J, K, L)**: The diameter of the largest liver lesion located in the right anterior lobe and segment 6 significantly decreased from 11.5cm to 7.7cm after six cycles of treatment. There was no markedly enhancement within the tumor during the delay phase.

A 46-year-old male with a history of chronic hepatitis B and hypertension was admitted to our hospital for the treatment of multiple liver tumors in December 2020. The liver tumors were detected at a local hospital after he presented with abdominal pain and abdominal distension for one month. The patient reported no past or present alcohol consumption or a history of diabetes. His family history and medical history were unremarkable. A physical examination of the patient showed no obvious positive signs. Laboratory investigations of liver and renal functions were all normal. The only abnormal laboratory results were carbohydrate antigen 19-9 (CA19-9), which was elevated to 710 U/ml. The levels of AFP, PIVKA-II and CEA were 2.77 ng/mL, 20 mAu/mL and 1.39 ng/mL, respectively. The indocyanine green retention rate at 15 min was 6.3%. The Child-Pugh Score was A with 5 points. Multiple liver tumors with ring-like enhancement were detected on abdominal contrast-enhanced CT and MRI. The largest lesion (measuring 11.5 cm in diameter) was located in the right anterior lobe and segment 6 (**Figure 1**). The hilar and retroperitoneal area revealed a shadow of multiple enlarged lymph nodes. No portal or inferior vena cava vein invasion or distant metastasis was observed. To rule out the possibility of liver metastases from gastrointestinal tumors, gastrointestinal endoscopy and colonoscopy were performed. No evidence of advanced malignant tumors was found. Considering that multiple intrahepatic tumors were unresectable, we performed an ultrasound-guided fine-needle aspiration biopsy to confirm the diagnosis. Pathohistological analysis of the tumor revealed adenocarcinoma arising from the biliary tract. The results of immunohistochemical analysis were as follows: PCK(+), EMA(+), CK7 (+), CK19 (+), AFP (-), Hepatocyte (-), VILLIN (+), Glypican-3(-), Arginase-1(-), CK20(-), CD34 (-), and Ki-67 labeling index: 30%. Based on these findings we made a diagnosis of ICC with lymph node metastasis and without macrovascular invasion. The TNM clinical stage was determined as T2N1M0 (stage IIIB) according to the American Joint Committee on Cancer (AJCC) Cancer Staging Handbook, 8th edition.

## Treatment

According to the opinion of the ICC multidisciplinary team (MDT) in our hospital and NCCN clinical practice guidelines in oncology hepatobiliary cancers, the patient was not a candidate for radical resection. For unresectable advanced ICC, gemcitabine and cisplatin combination chemotherapy is the standard first-line treatment, but the overall survival results remain disappointing. There is growing evidence that molecular targeted agents and immunotherapy may improve overall survival in patients with advanced ICC (9). After ICC MDT consultations and the patient’s informed consent, anti-PD immunotherapy (pembrolizumab, at a dose of 200 mg every three weeks) and lenvatinib (8mg/qd) combined with gemcitabine and cisplatin was considered as treatment for the patient, which was initiated in December 2020. Gemcitabine and cisplatin were administered intravenously on a 21-day cycle. Gemcitabine (1000 mg/m^2^) and cisplatin (25 mg/m^2^) were administered on Days 1 and 8 of each cycle. The patient was treated and monitored regularly. Blood count, liver, renal, thyroid, adrenal, and cardiac functions, and tumor markers were monitored every four weeks before each round of treatment. Adverse events were assessed and graded using the National Cancer Institute Common Terminology Criteria for Adverse Events v4.0.

**Figure 2:**
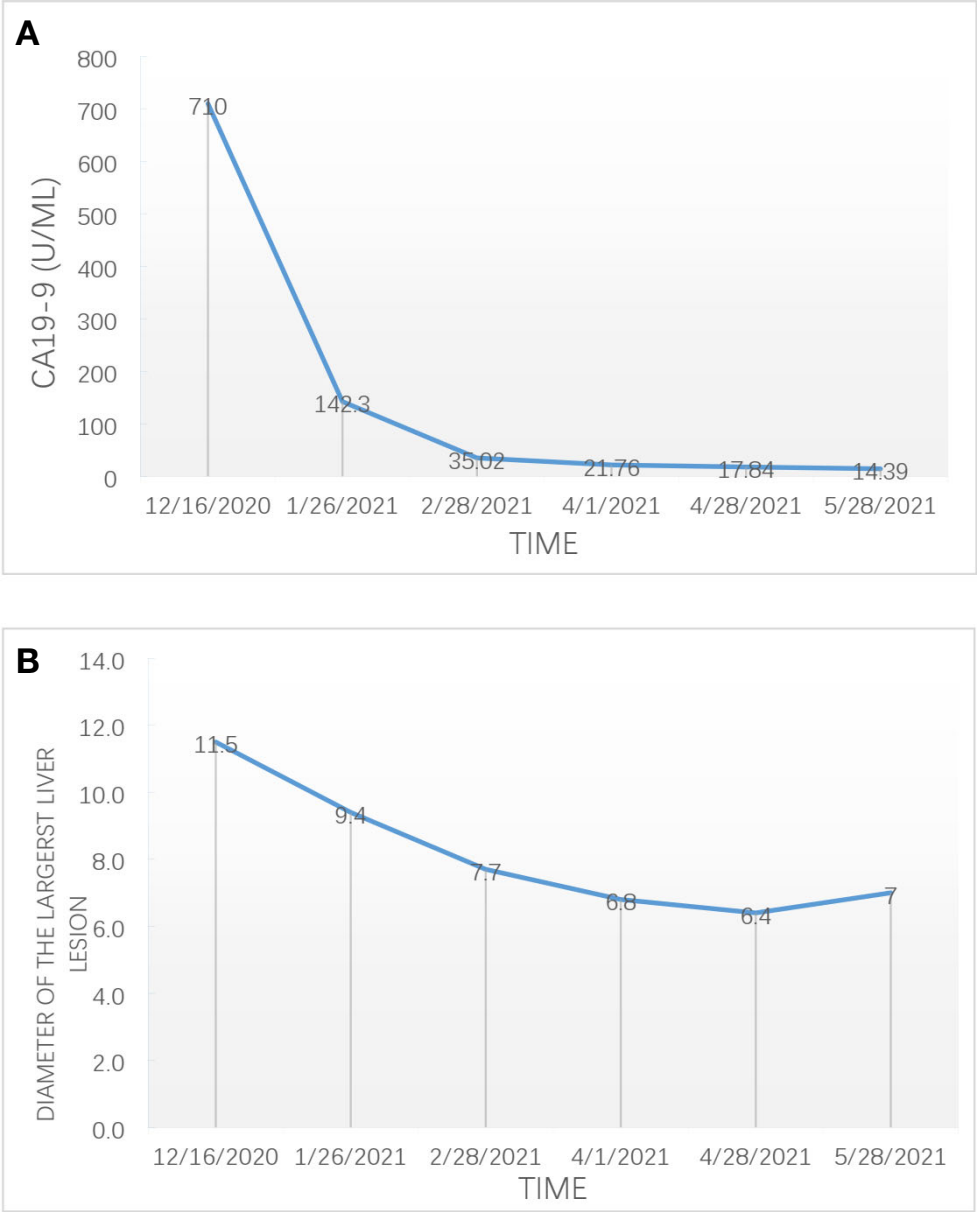
The change of the expression level of CA 19-9 **(A)** and the diameter of the largest liver lesion **(B)** during combination therapy for advanced ICC.

After three cycles of targeted therapy, immunotherapy and chemotherapy, the diameter of the largest liver lesion significantly decreased from 11.5 cm to 7.7 cm. The tumor marker CA19-9 dramatically decreased from 710 to 35 U/ml (**Figure 2**). The tumor response was evaluated and considered as partial response (PR) in February 2021 according to the mRECIST criteria. After six cycles of scheduled treatment, enhanced MR indicated that there was no significant change in the size of the largest tumor. However, the number of liver lesions decreased. The treatment effect is indicated by extensive central tumor necrosis (**Figure 1**). The level of the tumor marker CA19-9 demonstrated a constant downward trend with complete normalization (14.39 U/ml) by the end of his six cycles of treatment (**Figure 2**). A partial response was achieved without severe toxicities. The treatment-related adverse events were decreased appetite, neutrophil count decrease, platelet count decrease, white blood cell count decrease and diarrhea. All these immune-related adverse events were well controlled and alleviated after treatment. No intolerable grade 3–4 treatment-related adverse events occurred. Follow-up PET-CT revealed no metastasis and demonstrated the absence of hypermetabolic liver lesions and lymph nodes in the abdomen. One month after stopping the medication, we performed non-anatomical liver resection (segment 4b, 5,6 + segment 7 + segment 8), cholecystectomy and hilar lymph node dissection in July 2021.

## Operative findings

**Figure 3:**
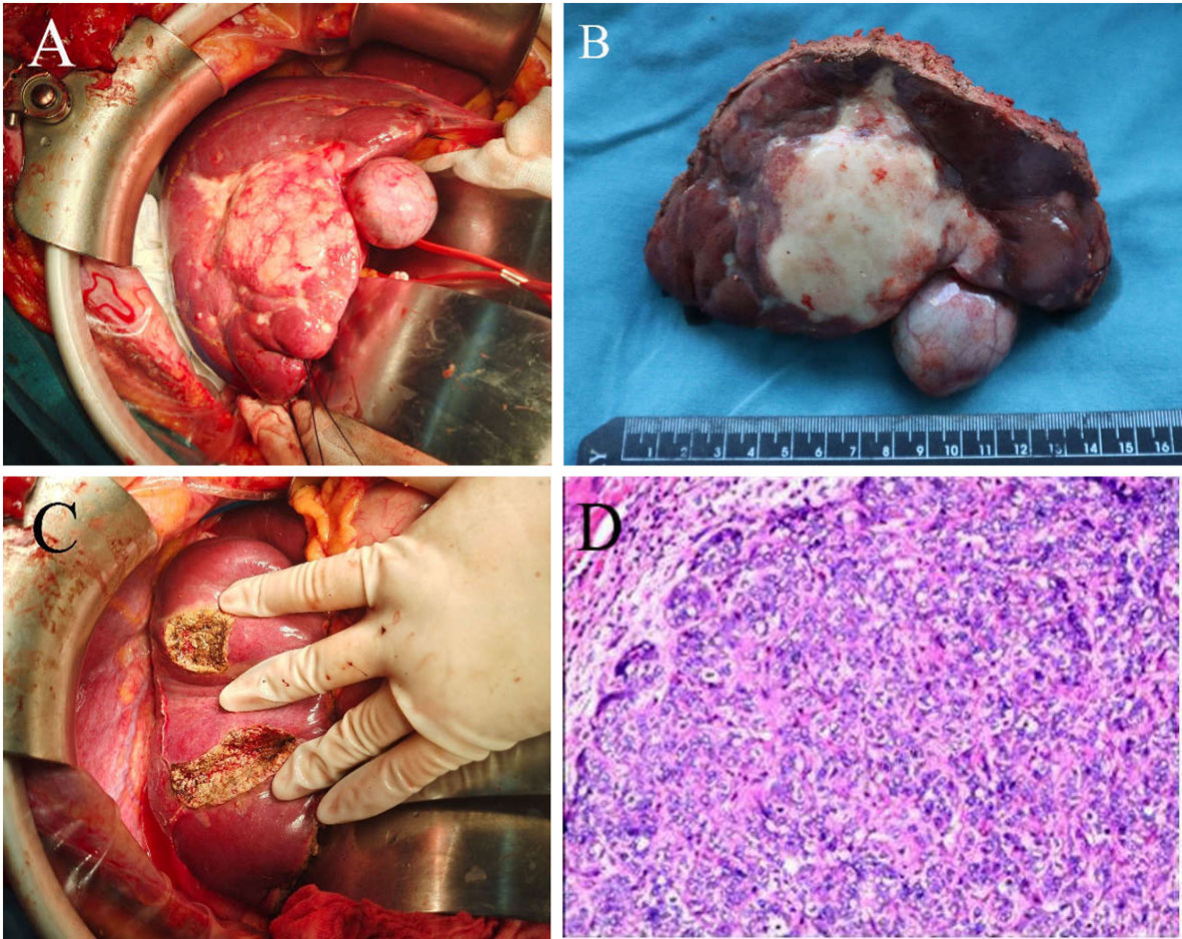
Intraoperative gross pathological specimen and H&E staining of resected specimen of a patient with advanced ICC who underwent radical liver resection. **(A)** and **(B)** A hard mass with a size of 7.5 x 5.3 cm was observed on the segment 4b, 5,6 of the liver, which was closely adjacent to the gallbladder. There were multiple satellite nodules surrounding the tumor. **(C)** Two lesions in segments 7 and segment 8 were completely resected. **(D)** H&E staining of resected tumor samples revealed predominately necrosis (>95%), hyaline change and cholesterol deposits.

Exploratory laparotomy with a reverse L-shaped incision in the right upper quadrant was performed. A hard mass with a size of 7.5 x 5.3 cm was observed on segment 4b, 5, and 6 of the liver, which was closely adjacent to the gallbladder. Multiple satellite nodules surrounding the tumor were observed. Intraoperative ultrasound identified two other lesions in segments 7 and 8. The maximal diameters of these two lesions were 1.4 and 1 cm, respectively (**Figure 3**). To ensure a wide (>1 cm) resection margin, intraoperative ultrasonography was used to delineate the cutting line. Parenchymal transection was performed by using a harmonic scalpel. Divided small diameter vessels were bipolar electrocoagulated, while larger vessels and bile ducts were transected after clamping with Hem-o-loks (Weck Surgical Instruments, Teleflex Medical, Durham, NC, USA) or with Endoscopic Rotating Multiple Clips (Ethicon Endo-Surgery). After the liver tumors were resected, residual bleeding sites were controlled with suture ligation or electrocautery. A silicone drain was applied to the raw liver transection plane.

## Outcome and follow-up

**Figure 4:**
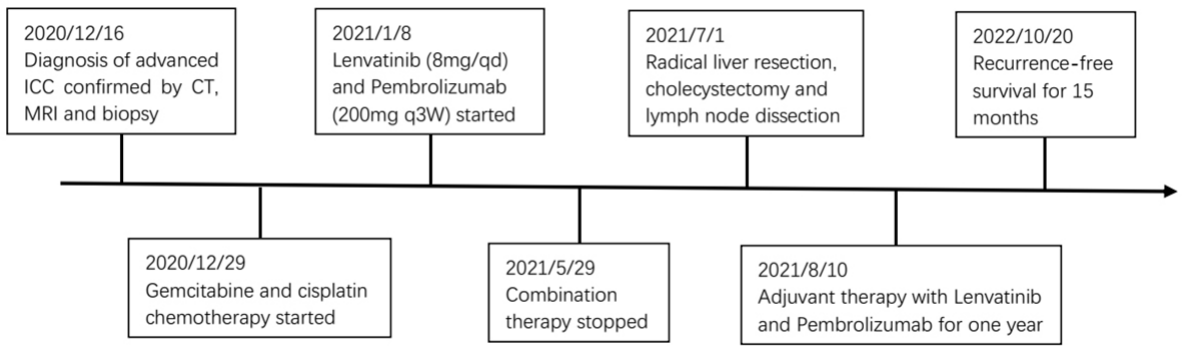
Timeline of initial diagnosis, combination therapy, radical surgery, adjuvant therapy and follow-up.

Pathological examination confirmed a diagnosis of moderate-to-poorly differentiated ICC with lymph node metastasis and without macrovascular and microvascular invasion. The resection margin was negative. H&E staining of resected tumor samples revealed predominant necrosis (>95%). The postoperative course was uneventful, and the patient was discharged 14 days after the surgery. Postoperative lenvatinib and pembrolizumab were continued for one year according to the usual dose and pattern. Follow-up was carried out at two-month intervals during the first year. Follow-up appointments consisted of history and physical evaluation, cell blood count, blood biochemistry, tumor markers, ultrasonography or enhanced CT. To date, the patient has survived 15 months following resection of the tumors, with no evidence of local recurrence or distant metastasis. **Figure 4** shows this patient’s timeline of initial diagnosis, combination therapy, radical surgery, adjuvant therapy and follow-up.

## Discussion

Intrahepatic cholangiocarcinoma is a highly malignant biliary tumor with poor outcomes. Early diagnosis and curative surgical resection remain the only options for long-term survival in patients with ICC. However, the rate of early diagnosis of ICC is low, and most patients (60%-70%) have lost their chance of surgery at the time of diagnosis (4). Even if patients undergo successful surgical resection, the high postoperative recurrence rate leads to only a 5-year survival of 20 to 40% in patients with ICC (16). Patients with unresectable BTC generally survive for less than 12 months after diagnosis (17). The combination of gemcitabine and cisplatin is currently the first*-*line treatment for patients with advanced BTC (6). Due to primary or acquired resistance to chemotherapeutic treatment, the median overall survival (OS) remains poor. Therefore, novel effective therapeutic approaches with distinct mechanisms of action are required for BTC. In recent years, immunotherapy has become the most promising approach for cancer therapy (18).

Multiple studies have evaluated the efficacy of immune checkpoint inhibitors as monotherapy in BTC. To date, KEYNOTE-158 is the largest study of ICIs monotherapy with pembrolizumab (19). Among 104 patients with advanced biliary cancers after progression or intolerance to standard therapy, the confirmed ORR was 5.8%; the median duration of response (DOR) was not reached (6.2-26.6 months). The median OS and progression-free survival (PFS) were 7.4 and 2.0 months, respectively. The anticancer activity of nivolumab has been evaluated in 54 patients with unresectable or metastatic cholangiocarcinoma or gallbladder cancers (20). Among the 46 enrolled patients, investigator-assessed PR and stable disease (SD) were achieved by 10 patients (22%) and 17 patients (32%), with a consequent DCR of 59%. Four responders (40%) achieved a durable objective response lasting at least one year. The mPFS was 3.68 months, and the mOS was 14.24 months. Although PD-1 blockade was recommended in the new NCCN guidelines for unresectable and metastatic ICC patients with dMMR or MSI-H, the limited antitumor effect of ICIs monotherapy in advanced cholangiocarcinoma has prompted investigators to explore different combination therapy strategies.

Evidence has accumulated that combined therapeutic strategies, including ICIs in combination with immunotherapy, chemotherapy, anti-angiogenics, or local therapy, improve the treatment efficacy over ICIs alone. In biliary tract cancer, durvalumab is being investigated in phase I studies in Asian patients (21). The results showed the superiority of the durvalumab–tremelimumab combination versus durvalumab alone in terms of OS (10.1 vs. 8.1 months) and DCR at 12 weeks (32.2% vs. 16.7%). Gemcitabine and cisplatin plus durvalumab for patients with advanced BTC have been evaluated in the phase 3 TOPAZ-1 study (22). A total of 685 patients with inoperable, locally advanced, recurrent, or metastatic BTC were randomly assigned to receive durvalumab or a placebo. OS and PFS were significantly improved in the durvalumab group. ORR was 26.7% for Durvalumab and 18.7% for placebo. Local tumor destruction combined with immunotherapy may have a synergetic effect against solid tumors (23). In addition to reducing the tumor burden, radiotherapy can lead to the triggering of antitumor immunity and the reprogramming of the tumor microenvironment (24). Zhao Q et al. (25) reported four cases of refractory advanced ICC or hilar cholangiocarcinoma effectively managed with anti-PD-1 antibody treatment following or concomitant with stereotactic body radiation therapy (SBRT). The median OS was greater than one year. Radical surgical resection was performed in one patient with a large lesion (12.9 × 11.8 cm) and multiple satellite lesions who was initially deemed unresectable. Thermal ablation therapy has shown remarkable local tumor control as a minimally invasive option for patients with unresectable hepatic malignancies, including cholangiocarcinoma (26). Xie C et al. (27) explored the efficacy of the combination of tremelimumab and microwave ablation in 20 patients with advanced BTC. An ORR of 12.5% and a DCR of 50% were observed. The median PFS, TTP, and OS were 3.4 months, 3.3 months and 6.0 months respectively. The combination treatment was well tolerated, with less than 10% of patients experiencing grade 3-4 toxicity. The outcomes of representative studies on the combination of ICIs with immunotherapy, chemotherapy, anti-angiogenics or local therapy in advanced BTC are summarized in **Table 1** (14, 21, 22, 27–41).

**Table 1:** Selected published trials on the combination of ICIs with immunotherapy, chemotherapy, anti-angiogenics or local therapy in advanced biliary tract cancer.

Author	Country	Trial number	Phase	Treatment Arm(s)	Patients	Line of treatment	ORR (%)	PFS (Months)	OS (Months)	Ref
ICIs in combination with immunotherapy										
Ioka T	Japan	NCT01938612	I	Durvalumab (D) +/− Tremelimumab (T)	D (n= 42) D+T (n = 65)	Second	4.8 in D, 10.8 in D+T	–	8.1 in D, 10.1 in D+T	(21)
Klein O	Australia	CA209-538	II	Nivolumab + Ipilimumab	39	First and Second	23	2.9	5.7	(28)
Kelley RK	US	NCT02703714	II	Pembrolizumab+GM-CSF	27	Second	21	–	–	(29)
ICIs in combination with chemotherapy										
Ueno M	Japan	JapicCTI-153098	I	Nivolumab vs Nivolumab + GP chemotherapy	30 vs 30	Second	3 vs 37	1.4 vs 4.2	5.2 vs 15.4	(30)
Feng K	China	NCT03311789	II	Nivolumab+GP chemotherapy	27	First and Second	55.6	6.1	8.5	(31)
Sahai V	US	NCT03101566	II	Nivolumab+GP chemotherapy vs Nivolumab + ipilimumab	35 vs 33	First	22.9 vs 3	6.6 vs 3.9	10.6 vs 8.2	(32)
Oh DY	Korea	TOPAZ-1 NCT03875235	III	Durvalumab+GP chemotherapy vs placebo + GP chemotherapy	341 vs 344	First	26.7 vs 18.7	7.2 vs 5.7	12.8 vs 11.5	(22)
Li W	China	NCT03796429	II	Toripalimab+ gemcitabine+S1	50	First	30.6	7	15	(33)
Chen X	China	NCT03486678	II	Camrelizumab+GEMOX	38	First	80 in PD-L1 TPS≥1%, 53.8 in TPS < 1%	6.1	11.8	(34)
Chen X	China	NCT03092895	II	Camrelizumab+GEMOX or FOLFOX	92	First	16.3	5.3	12.4	(35)
ICIs in combination with anti-angiogenics										
Arkenau HT	UK	NCT02443324	I	Pembrolizumab +Ramucirumab	26	Second	4	1.6	6.4	(36)
Lin J	China	NCT03892577	Real-world Study	Pembrolizumab or nivolumab + lenvatinib	56	First	30.5	5	11	(37)
Villanueva L	International Multicenter	LEAP-005 NCT03797326	II	Pembrolizumab + Lenvatinib	31	Second	10	6.1	8.6	(38)
Zhang Q	China	ChiCTR2100044476	II	PD-1 inhibitors + Lenvatinib	38	First	42.1	8	17.7	(39)
Shi C	China	–	Retrospective study	PD-1 inhibitors + Lenvatinib	74	Second	20.27	4	9.5	(14)
Jian Z	China	NCT03951597	II	Toripalimab + lenvatinib + GEMOX chemotherapy	30	First	80	10	–	(40)
ICIs in combination with local therapy										
Hong TS	US	NCT03482102	I	Durvalumab-tremelimumab + radiotherapy	15	Second	20	1.8	–	(41)
Xie C	US	NCT01853618	II	Tremelimumab + microwave ablation	16	Second	12.5	3.4	6	(27)

There are few reports on the efficacy of the combination of angiogenesis/checkpoint blockade with chemotherapy in advanced ICC. In a recent phase II trial, the combination of toripalimab and lenvatinib plus gemcitabine and oxaliplatin (GEMOX) was evaluated as a first-line therapy for locally progressed or metastatic ICC (40). The ORR was 80% (24/30), and the DCR was 93.3% (28/30) among the 30 enrolled patients. CR was observed in one patient. Three patients with locally advanced disease received resection after being successfully downstaged. The median PFS was 10.0 months, and the median DOR was 9.8 months. In this case, we adopted similar combination treatment strategies for a patient with stage IIIB ICC. To our knowledge, this is the first case report describing a patient with unresectable ICC who received conversion therapy with lenvatinib, pembrolizumab and GP chemotherapy and subsequently underwent radical liver resection.

The combination treatment has significant antitumor activity. After six cycles of treatment, the patient with initial unresectable ICC finally received radical resection without recurrence or distant metastasis after 15 months of follow-up. The possible mechanisms of the synergistic antitumor effect of combination therapy with ICIs, antiangiogenic agents, and chemotherapy may include the following: First, chemotherapy may improve the effectiveness of immunotherapy by reducing the microenvironment’s immunosuppressive effects, increasing the cross-presentation of tumor antigens, and facilitating immune cell infiltration into the tumor core (42–44). Second, antiangiogenic therapy has several immunostimulatory effects, including an increase in the trafficking of T cells into tumors and a decrease in immunosuppressive cytokines and T regulatory cells (45–47). We found that the tumor marker CA19-9 dramatically decreased after only three cycles of treatment. The results of previous studies indicated that a decrease in plasma tumor markers after lenvatinib treatment was an important predictor of successful R0 resection for advanced HCC (48).

With the development of systemic therapy, especially immunotherapy, there has been a growing number of reports of unresectable BTCs being converted into resectable ones followed by surgical removal of the tumors.

A recent systematic review including patients with unresectable ICC from 10 trials indicated a surgical conversion rate of 17.3% (27/132) with the use of various therapeutic modalities (49). Miyamoto R et al. (50) reviewed the retrospective studies about conversion surgery with chemotherapy for initially unresectable BTC. The conversion rate ranged from 0.65 to 80%, and the R0 resection rate ranged from 4.8 to 80%. In a phase 2 (MISPHEC) trial, Edeline J. et al. (51) reported that 22% of patients with advanced ICC receiving radioembolization plus chemotherapy could be downstaged to surgical intervention, of which 8 patients survived after 24 months of follow-up. By means of systemic or regional therapy, adequate tumor downstaging was accomplished, and patients could obtain a generally favorable prognosis (52–54). Le Roy B. et al. (55) reported the short- and long-term outcomes of patients with advanced ICC treated with surgery after chemotherapy were comparable to those of patients with initially resectable ICC treated with surgery alone. Recently, an open-label phase II trial studied the efficacy of combining lenvatinib with a PD-1 inhibitor for the treatment of advanced BTC for the first time (39). Thirty-eight patients were enrolled, with an ORR of 42.1%, and a DCR of 76.3%. Thirteen (34.2%) achieved downstaging and underwent surgery, and six of them (46.2%) achieved a pathologic response. In order to augment the response rate and increase the proportion of patients downstaged to resectability, prospective studies comparing efficacy are needed to evaluate the optimal conversion therapy strategies for advanced ICC.

In our study, Lenvatinib and Pembrolizumab in combination with GP chemotherapy had a manageable safety profile and was well tolerated. The main treatment-related adverse events were haematological toxicities. In the ABC-02 randomized phase 3 study comparing gemcitabine plus cisplatin with gemcitabine alone in patients with advanced BTC, the rate of any hematologic toxic effect was 32.3% in the GP group and 23.6% in the gemcitabine alone group (6). In a single-arm, phase II trial including 50 patients with advanced BTCs, toripalimab in combination with GP chemotherapy showed encouraging efficacy and safety (33). Chemotherapy combined with immunotherapy did not increase hematological toxicity compared to chemotherapy alone. Jian Z et al. (40) reported that Grade 3 or 4 neutropenia and thrombocytopenia observed in 10% and 3.3% patient who received toripalimab, lenvatinib in combination with GEMOX chemotherapy for advanced ICC.

## Conclusion

In conclusion, we reported a case of initially unresectable and advanced ICC that was successfully downstaged to surgical curative-intent treatment after receiving lenvatinib and pembrolizumab combined with GP chemotherapy. The findings suggested that this combination therapy was a potentially feasible and safe conversion therapy strategy for these patients. The dynamic change in the level of tumor markers might be an approach for the detection of tumor response. The exact safety and efficacy of antiangiogenic TKI and anti-PD-1 antibodies combined with chemotherapy in patients with advanced BTC need to be compared with chemotherapy alone in a prospective controlled study.

## Data availability statement

The original contributions presented in the study are included in the article/supplementary material. Further inquiries can be directed to the corresponding author.

## Ethics statement

Ethical review and approval was not required for the study on human participants in accordance with the local legislation and institutional requirements. The patients/participants provided their written informed consent to participate in this study. Written informed consent was obtained from the individual(s) for the publication of any potentially identifiable images or data included in this article.

## Author contributions

WZ contributed to design of work. Z-YZ, B-XZ and X-PC contributed to acquisition, analysis, and interpretation of data. WZ and CL contributed to drafting and revising. All authors were accountable for all aspects of the work and approved the final version.
